# Upregulation of PKN1 as a Prognosis Biomarker for Endometrial Cancer

**DOI:** 10.1177/10732748221094797

**Published:** 2022-05-09

**Authors:** Igor Govorov, Sanaz Attarha, Larysa Kovalevska, Emil Andersson, Elena Kashuba, Miriam Mints

**Affiliations:** 1 Department of Women’s and Children’s Health, Division of Obstetrics and Gynecology, Karolinska University Hospital, Solna, 27106 Karolinska Institutet, Stockholm, Sweden; 2 Science for Life Laboratory, 27106 Karolinska Institutet, Stockholm, Sweden; 3 123495 R. E. Kavetsky Institute of Experimental Pathology, Oncology and Radiobiology of NASU; 4 Department of Microbiology, Tumor and Cell Biology (MTC), Biomedicum, 27106 Karolinska Institute, Stockholm, Sweden; 5 School of Medical Science, Faculty of Medicine and Health, 6233 Örebro University, Örebro, Sweden

**Keywords:** endometrial cancer, PKN1, prognostic marker, tumor progression, survival

## Abstract

**Background:**

Several markers of survival among endometrial cancer (EC) patients have been proposed, namely, the oncoprotein stathmin, RAF kinase inhibitor (RKIP), Cyclin A, GATA-binding protein 3 (GATA3), and growth and differentiation factor-15 (GDF-15). Their elevated expression correlated significantly with a high stage, serous papillary/clear cell subtypes, and aneuploidy. In a previous study, we reported the elevated expression of the serine/threonine protein kinase N1 (PKN1) in cancerous cells. In the present paper, we studied PKN1 expression in EC tissues from a large cohort of patients, to determine whether PKN1 can serve as a marker for the aggressiveness and prognosis of EC, and/or as a marker of survival among EC patients.

**Methods:**

Tissue samples from EC patients were examined retrospectively for tumor type, tumor size, FIGO stage and grade, depth of invasion in the myometrium, and presence of lymph node metastasis. The PKN1 protein expression in EC cells was assessed by immunohistochemistry. *PKN1* mRNA levels were analyzed in publicly available databases, using bioinformatic tools.

**Results:**

We found that expression of *PKN1* at the mRNA and proteins levels tended to increase in high-grade EC samples (P = .0001 and P = .06, respectively). In addition, patients with metastatic disease had higher PKN1 mRNA levels (P = .02). Moreover, patients with high *PKN1* expression could be characterized by poorer survival.

**Conclusions:**

We have shown a trend of the higher *PKN1* expression levels in EC patients with poor prognosis. Therefore, *PKN1* might be considered as a candidate prognostic marker for EC.

## Introduction

Endometrial cancer (EC) is the most common gynecological malignancy in high-income countries. The incidence rate of EC rises worldwide due to the overall aging of the population and the increased burden of risk factors. The latter is usually referred to unopposed estrogen exposure, both endogenous and exogenous. This embraces obesity, early menarche and late menopause, the late date of childbirth, polycystic ovary syndrome, estrogen-only menopausal hormone therapy (MHT), use of tamoxifen, etc.

EC usually presents at an early stage with abnormal uterine bleeding and therefore has a favorable prognosis. However, even in patients without metastatic disease, the 5-year survival fluctuates significantly, from 74 to 91%.^1^ This indicates a more significant heterogeneity of these tumors than is accepted in the conventional dichotomous classification: type 1 and type 2. The latter was initially proposed by Bokhman in his landmark study back in 1983^2^ with type 1 representing endometrioid tumors, and type 2—serous. Type 2 later included clear cell carcinomas and Grade 3 endometrioid tumors. The new emerging approaches aim to classify patients into one of four groups, depending on tumor molecular characteristics.^3^ Currently, active research is underway to investigate the potential molecular biomarkers that would allow stratifying patients with EC into subgroups, guiding the appropriate management and predicting the ultimate prognosis.^4^

Hormone receptor expression, primarily receptors of estrogen (ER) and progesterone (PgR), are well-known prognostic factors with its positivity contributing to a more prolonged disease-free survival.^5^ Other markers under current focus include phosphatase and tensin homolog *(PTEN)* and wild-type *TP53* genes, and the P13 K/AKT/mTOR pathway.^6-8^

One of the putative markers, that can be a molecular target in the future, is a member of the protein kinase C superfamily—the serine/threonine-protein kinase N1 (PKN1, NP_998 725), also known as protein kinase C-related kinase 1 and PKN-alpha. Previously, it was reported that in cancerous cells, this gene showed elevated expression and proposed *PKN1* expression as a putative predictive biomarker for the course of EC.^9,10^ It was shown later on, that, indeed, PKN1 expression was elevated in cancerous cells, as a rule.^11-13^ Importantly, PKN1 plays a role in functioning of the intermediate cytoskeleton filaments, cell migration, and, as a consequence, in tumor invasion.^14^ In the current study, we evaluated the utility of PKN1 as a putative prognostic marker in patients with EC on a large cohort of patients.

Therefore, the primary aim of the current study was to determine whether PKN1 can serve as a marker of aggressiveness, prognosis, and survival among patients with EC, using tissue samples and publicly available databases.

## Materials and Methods

### Study Cohort

Ninety-five patients with endometrioid EC, four with serous EC, and one with carcinoma with no prior chemotherapy or radiation therapy were included. Tissue samples were collected together with relevant anthropometric and clinical data.

### Ethical Permission

This study was approved by the Ethical Committee (protocol number 3 from 25th of June 2019). Written informed consent was obtained from all patients and all protocols were performed in accordance with the ethical regulations.

### Histological Evaluation

Tissue samples were fixed in a neutral buffered 4% formaldehyde solution. After fixation, dehydration, and embedding in paraffin, serial sections were cut at a standard thickness of 5 *μ*m and stained with hematoxylin/eosin for histological diagnosis. All tissues were examined by 2 experienced gynecologic pathologists independently, and they determined tumor type, tumor size, FIGO stage (I-IV), FIGO grade (1-3), depth of the myometrial invasion,^15^ lymph node metastasis, and sex steroid expression.

### Immunohistochemistry

PKN1 expression in EC cells was assessed by immunohistochemistry. Briefly, paraffin-embedded tissue samples were heated for 15 min at 55°C. Paraffin was removed by dissolution in xylene with the following wash with ethanol (99%, 70%, and 30% sequentially). Tissue samples were then treated by a 2% solution of H_2_O_2_ in methanol at room temperature for 30 min to reduce background staining. After re-hydratation, antigens were retrieved in citrate buffer by heating (water bath, 92°C for 15 min). Tissue samples were stained with the primary anti-PKN1 antibody (H-234, Santa Cruz Biotechnology Inc., Santa Cruz, CA, USA) at a dilution of 1:100 in blocking buffer (2% bovine serum albumin, .2% Tween-20, 10% glycerol, and .05% NaN_3_ in phosphate-buffered saline). Next, the immunofluorescence staining was run with Crystal violet; DAPI counterstaining, after the secondary swine anti-rabbit FITC-conjugated antibody (DAKO, Glostrup, Denmark) was applied. Imaging and an image analysis were performed, as described earlier.^16^ Staining was evaluated manually, counting the PKN1-positive EC cells. The minimum number of tumor cells we found in any EC tissue sample was 900.

### Statistical Analysis

GraphPad Prism software (version 8, GraphPad Software, La Jolla, CA, USA) was used to perform multiple comparisons of non-parametric criteria. The means of the PKN1 expression (as a per cent of cells that were positive for PKN1 expression) were analyzed. Patients were then categorized by FIGO stage, FIGO grade, and further analysis was performed on the combined mean of each set of tumors, according to these parameters. A detailed description of the calculations is given in the figure legends. Briefly, the Kruskal–Wallis test was performed to determine differences in PKN1 expression across categories of FIGO stage, FIGO grade, and selected clinical characteristics (age, ER and PgR expression, and BMI). To evaluate the putative dependence between PKN1 expression and survival, only EC-specific survival was assessed.

To further strengthen the results of PKN1 expression patterns and survival analysis in our 95 EC patients, we also analyzed data on mRNA expression of PKN1 in 54 patients with EC from the Oncomine database. This database is publicly available and contains published data that has been collected, standardized, annotated, and analyzed by Compendia Bioscience (www.oncomine.com, March 2021, Thermo Fisher Scientific, Ann Arbor, MI, USA). The quantitative data on PKN1 mRNA levels were retrieved from the Oncomine database and analyzed using the non-parametric methods. Protein and mRNA expression were assessed together in the context, although not directly tested for correlation due to different data sources.

Additional confirmatory analyses were performed using data from the Human Protein Atlas.^17^ In all analyses, the significance threshold was set at the level of P <.05.

## Results

### Patient and Tumor Characteristics

The patients in the studied cohort were predominantly elderly people, with the mean age 71, slightly overweight and with no prior history of diabetes. The majority presented at an early stage, and few died of EC relapse. The ultimate background and clinical characteristics are presented in Table 1.

**Table 1.** Background and clinical characteristics of the study group.

		Median (25–75 Percentile)	Min/Max
**Age, years**		71.0 (67.0–77.0)	36/92
**BMI**		26.3 (23.8–29.5)	19.0/40.0
**Progesterone receptor expression, %**		70.0 (28.8–90.0)	0/100
**Estrogen receptor expression, %**		80.0 (48.8–95.0)	0/100
		n*	
**MHT**	Yes	45	
	No	49	
**Diabetes**	Yes	10	
	No	87	
**Tumor type**	Endometrioid	95	
	Non-endometrioid	5	
**Stadium**	I	68	
	II	18	
	III	13	
	IV	1	
**Grade**	1	28	
	2	41	
	3	31	
**Myometrial invasion**	None	4	
	≤50%	46	
	≥50%	49	
	Through serosa	1	
**TP53 expression**	Negative	86	
	Positive	11	
**Ploidy**	Aneuploid	33	
	Diploid	65	
**Adjuvant therapy**	None	23	
	Radiation	54	
	Chemo	2	
	Radiation + chemotherapy	20	
**Died of EC**	Yes	19	
	No	81	

*Since the total number of participants is 100, the number equals the percentage.

**Figure 1.**
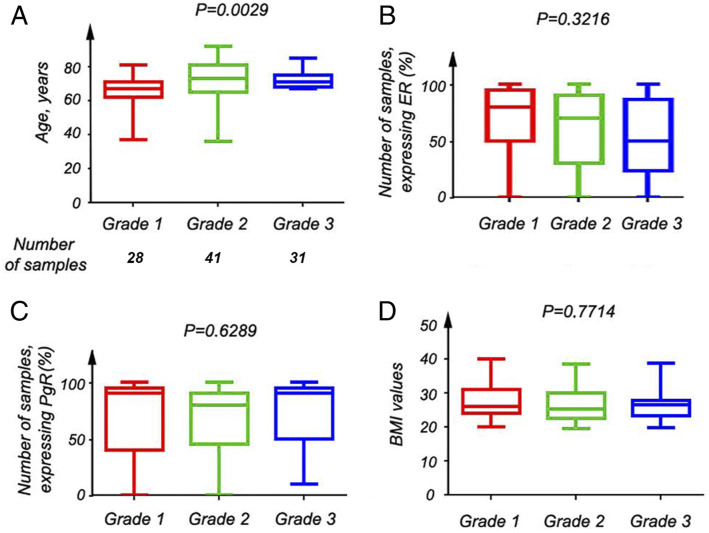
Clinico-pathological characteristics of EC patients. (A) A significant age increase was observed for patients with more developed EC, according to the Kruskal–Wallis test of three different groups; patients were grouped by grade, that is, 1–3. (B) The Kruskal–Wallis of 3 groups of patients, divided by the tumor grade showed a tendency of ER to decrease upon tumor progression. (C) No significant differences were calculated for PgR in 3 groups of patients, according to the Kruskal–Wallis test, when they were divided by the tumor grade. (D) The Kruskal–Wallis test was performed to monitor the body mass index (BMI) values in patients, grouped, accordingly to 1–3 tumor grade. No difference was observed, though.

When looking at the FIGO stage, patients with more advanced EC tended to be older; this was also true when looking at FIGO grade (P = .0029) (Figure 1A). A trend of decreasing ER expression with the increasing of FIGO grade was also observed (Figure 1 B). However, no significant trend was found for PgR expression or BMI (P = .63 and P = .77, respectively) (Figure 1C and D).

### Expression Pattern of PKN1 at the Protein and mRNA Levels

**Figure 2.**
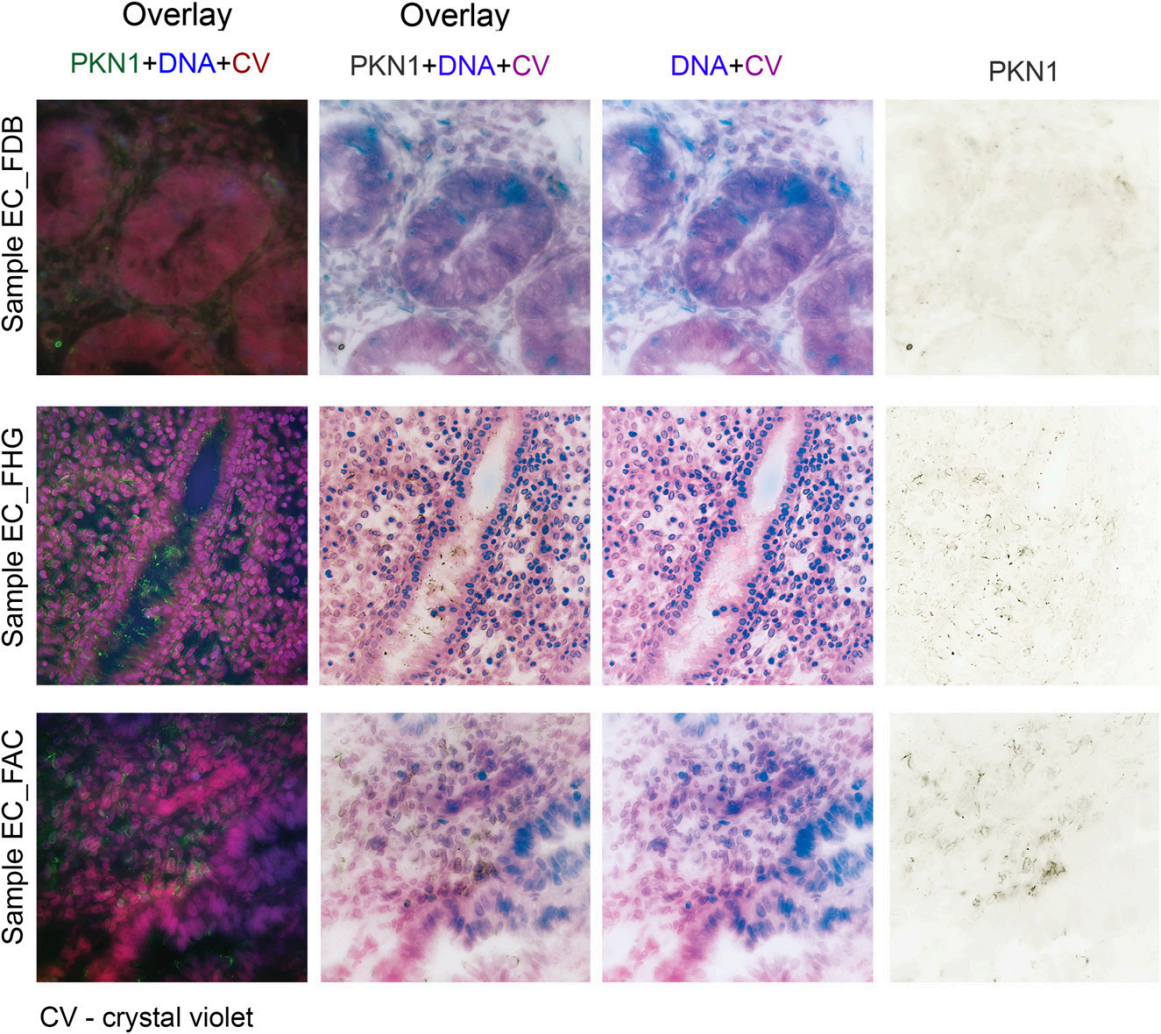
Expression pattern of the PKN1 protein in EC samples. Expression of PKN1 was assessed by fluorescent microscopy, using the specific antibody. The strong signal (shown in dark brown) was detected mainly in cytoplasm (see the right column). Notice the increase in intensity of PKN1 signal with a higher tumor grade (EC_FDB – Grade I, EC_FHG – Grade 2, EC_FAC – Grade 3). Nuclei are shown in blue. Tissue architecture is shown in red (crystal violet, CV).

**Figure 3.**
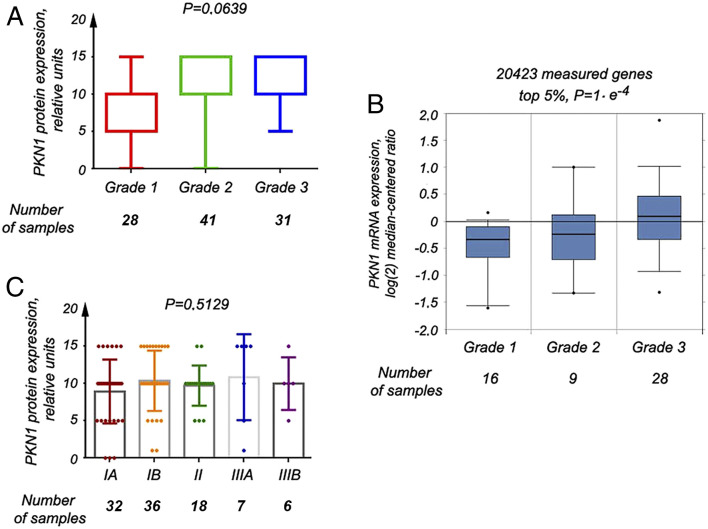
PKN1 expression at the mRNA and protein levels in different groups of EC patients. (A) The PKN1 protein expression was elevated with the increasing of tumor grade, according to the Kruskal-Wallis test of 3 different groups. (B) Similarly, the PKN1 mRNA expression raised with the tumor progression, as extracted from the Oncomine database. (C) No differences in PKN1 expression were revealed by the Kruskal–Wallis test for 5 groups, when samples were divided, according to the tumor stage.

Strong PKN1 signals were detected mainly in the cytoplasm of EC tissue samples (Figure 2). PKN1 expression increased along the higher FIGO grade (P = .06) (Figure 3A). This was corroborated by the data on *PKN1* mRNA expression from the Oncomine database (P = .0001) (Figure 3B). However, when our data was categorized by the FIGO stage, only slight non-significant (P = .51) increase in PKN1 expression was observed (Figure 3C).

### PKN1 Expression and Survival

**Figure 4.**
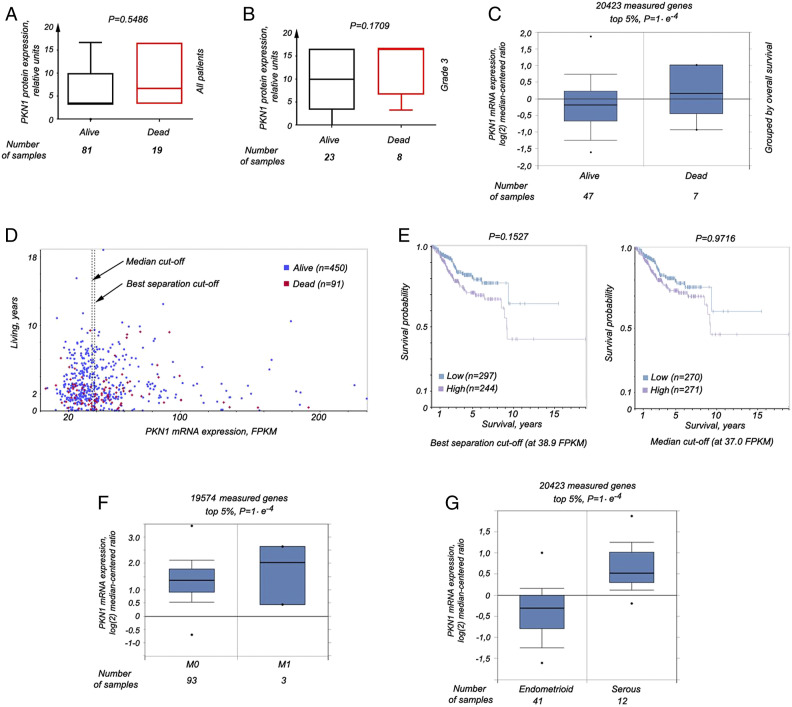
PKN1 expression levels correlate with a survival rate. (A) The Kruskal–Wallis test for 2 groups showed a trend for increase of the PKN1 protein expression in samples of EC of deceased patients. (B) This phenomenon was more obvious when samples of patients with grade 3 EC were compared in the same analysis. (C) Expression of PKN1 at the mRNA levels was significantly higher as well in samples of deceased individuals, as extracted from the data at the Oncomine portal (P = .0183). (D) The expression of the PKN1 gene at the mRNA level, as shown at the Protein atlas website. mRNA expression is presented in FPKM units, that is, fragments per kilobase million. Patients were divided into 2 groups, according to high and low PKN1 expression, using the median expression cut-off (at 37.0 FPKM) and also best separation cut-off (at 38.9 FPKM). These points are indicated by dotted lines and arrows. (E) - The Kaplan–Meier plot was built for each case (left and right panels correspond to the best separation and the median expression cutoffs). The long-rank P-values were calculated as well. (F) Expression of PKN1 at the mRNA levels was significantly higher in tumor samples of patients with metastases, as extracted from the data at the Oncomine portal (P = .0183). (G) In PKN, levels are significantly elevated in serous EC, as compared with such levels in endometrioid EC, according to the data at the Oncomine portal (P = .0183).

When PKN1 expression was compared in the tissue samples of living and deceased patients in our study sample, a trend of elevated PKN1 expression was observed in deceased patients (P = .55) (Figure 4A). A clearer picture was uncovered when looking at FIGO grade 3 tumors (P = .17) (Figure 4B). When we considered 54 patients from the Oncomine database, *PKN1* mRNA expression was also higher in the tissue samples of deceased patients (P = .0001) (Figure 4C). Additional confirmatory analyses performed on data from 541 samples in the Human Protein Atlas (450 living and 91 deceased individuals), divided into groups of high and low *PKN1* expression (Figure 4D), showed a survival curve that looked somewhat different, depending on the cut-off applied (Figure 4E). However, the common trend was the same—patients with high PKN1 expression in EC tissue samples had shorter survival than individuals with low *PKN1* expression, regardless of whether the groups were created based on median expression (Figure 4E, right panel) or best separation (Figure 4E, left panel).

In tumor samples of patients with metastases in the Oncomine database, high *PKN1* expression was also observed (Figure 4F, P = .0001). Furthermore, increased *PKN1* mRNA expression was found in serous tumors compared with endometrioid, according to the Oncomine (Figure 4G).

## Discussion

Reliable prognostic markers are essential to predict the course of the disease, but there are currently not many such markers for EC.

In the present work, we assessed the PKN1protein expression using laboratory methods and the expression of *PKN1* at the mRNA level, using bioinformatic methods. We then compared these expression patterns, together with clinic-pathological characteristics of the patients. We found increasing *PKN1* expression with higher FIGO grade in patients with endometrioid EC in both, our study samples, and confirmatory analyses. Tumor grade is used to describe how much cancer cells resemble the healthy counterparts, while tumor stage reflects the degree of tumor spread. Therefore, our results suggest that poorly differentiated endometrial tumors possesses higher PKN1 expression at both mRNA and protein levels. Moreover, higher *PKN1* expression was observed among patients with metastases and patients with more aggressive serous EC in confirmatory analyses, compared with patients without metastases and with type 1 tumors, respectively.

High levels of wild-type TP53 and presence of the mutated *PTEN* gene might serve as prognostic markers for endometrioid EC (type 1 EC), but no considerable progress has been made in this field, despite the development of powerful laboratory methods.^18,19^

Importantly, *PKN1* expression has been shown to be elevated in triple-negative breast cancer samples (i.e., when ER, PgR, and the avian erythroblastic leukemia viral oncogene homolog 2 (ERBB2, also known as Her2/neu) are downregulated or absent), that usually show a very aggressive phenotype.^11^ Therefore, we may conclude that the increase in *PKN1* expression we observed is associated with more aggressive tumor phenotypes. This could be due to the enhanced motility of cancerous cells and the evasion of apoptosis. As mentioned above, PKN1 can contribute to enhanced motility of tumor cells through interaction with proteins of the Ras homolog gene family (Rho) that are functioning as GTPases.^12^

In prostate cancer, another receptor-dependent tumor, it was shown that PKN1 is involved in the induction of prostatic epithelial neoplasia.^20^ Moreover, in hormone-dependent prostate cancer, PKN1 phosphorylation stimulates transactivation of androgen-dependent genes.^21^ The kinase activity of PKN1 is also required for androgen-independent prostate tumors to metastasize—the inhibition of PKN1 resulted in the prevention of metastases in mouse models.^22^

PKN1 can also inhibit the Wnt/beta-catenin signaling, especially in melanoma cells. It was demonstrated that diminishing PKN1 expression induced apoptosis in melanoma cells.^14^ Different PKN isoforms perform different functions depending on the tissue type.^23^ For example, the PKN3 and PKN1 isoforms play a significant role in prostate cancer development, and the same is true for the PKN2 isoform in bladder cancer. We plan to study PKN2 and PKN3 expression in EC in a future work. In summary, we may conclude that higher PKN1 levels in more aggressive tumors are associated with promoting metastasis and invasion.

Among the putative limitations of this work, we have to mention its retrospective nature, which has inherent weaknesses and lack of time-to-event data, preventing to bring additional information into the present work. On the other hand, we worked with a reasonably large cohort and performed a thorough immunohistochemical study and an analysis of available databases, to correlate the obtained experimental results with those available.

Could PKN1 expression be a prognostic marker as well as an indicator of survival? Our study sample showed an association (the trend) between high PKN1expression and poor prognosis. High expression of PKN1 was found in high-grade tumors, which demonstrate aggressive growth and high rate of spread. In addition, increased PKN1 expression was inherent in metastatic and serous tumors that are known to have worse prognosis. Importantly, our confirmatory analyses, carried out using data from external databases led us to the same conclusion—that overall survival was lower for patients with high PKN1 expression.

## Conclusions

Summarizing, we found that *PKN1* is highly expressed in high-grade, low-differentiated EC at the mRNA and protein levels, and expression is increasing with EC progression. Moreover, patients with high PKN1 expression could be characterized by poorer survival. Therefore, PKN1 is a strong candidate prognostic marker for EC.

## Appendix

### Abbreviations

BMI: body mass index
EC: endometrial cancer
ER: estrogen receptor
FIGO: the International Federation of Gynecology and Obstetrics
MHT: menopausal hormone therapy
mRNA: messenger RNA
PgR: progesterone receptor
PKN1: the serine/threonine-protein kinase N1
PTEN: phosphatase and tensin homolog
